# Circadian Timing, Rather than Hydration Status, Determines Metabolic Adaptation to Ramadan-like Fasting in Diet-Induced Obese Rats

**DOI:** 10.3390/nu18040663

**Published:** 2026-02-18

**Authors:** Noof M. Alshahrani, Maha H. Alhussain, Mohammed F. Alahmed, Ahmad T. Almnaizel, Ahmed S. BaHammam

**Affiliations:** 1 Department of Food Sciences and Nutrition, College of Food and Agricultural Sciences, King Saud University, Riyadh 11451, Saudi Arabia; n.shahrani09@gmail.com; 2 Prince Naif bin Abdulaziz Health Research Center, Riyadh 11461, Saudi Arabia; moalahmed@ksu.edu.sa; 3 Research Office, Johns Hopkins Aramco Healthcare, Dhahran 31311, Saudi Arabia; ahmad.almnaizel@jhah.com; 4 Department of Medicine, College of Medicine, King Saud University, Riyadh 11461, Saudi Arabia; ashammam2@gmail.com; 5 University Sleep Disorders Center, King Saud University Medical City, King Saud University, Riyadh 11461, Saudi Arabia; 6 The Strategic Technologies Program of the National Plan for Sciences and Technology and Innovation, Riyadh 11442, Saudi Arabia

**Keywords:** Ramadan fasting, circadian rhythm, obesity, metabolism, adipokines, hydration, rats

## Abstract

Background: Ramadan fasting involves daily abstinence from food and water between dawn and sunset, but human studies cannot readily disentangle the effects of fasting timing, circadian alignment, and hydration status on metabolic regulation. Objective: To determine whether fasting timing or hydration status exerts a stronger influence on metabolic outcomes in diet-induced obese rats under controlled Ramadan-like conditions. Methods: Forty diet-induced obese rats were assigned to four Ramadan-like fasting groups differing by timing and hydration: dry morning (DM), wet morning (WM), dry night (DN), or wet night (WN) fasting, in addition to healthy control (HC) and obese control (OC) groups (n = 8 each). Because rats are nocturnal, morning fasting restricted food during the inactive (light) phase, whereas night fasting restricted food during the active (dark) phase. Body weight, glucose, insulin, HOMA-IR, lipid profile, adipokines, and electrolytes were assessed after four weeks. Results: Morning fasting significantly reduced body-weight gain (F(5,42) = 10.72, *p* < 0.0001; η^2^ = 0.56) and improved insulin sensitivity, reflected by lower insulin (F(5,30) = 2.98, *p* = 0.027; η^2^ = 0.33) and HOMA-IR (F(5,30) = 3.76, *p* = 0.0092; η^2^ = 0.39), independent of hydration status. Serum glucose differed across groups (F(5,42) = 5.82, *p* = 0.00036). After body-weight adjustment, total cholesterol and triglycerides were reduced in fasting groups, whereas hydration primarily influenced fluid and electrolyte parameters without materially altering core metabolic outcomes. Conclusions: Under controlled conditions, fasting timing exerted a stronger influence on metabolic regulation than hydration status. Fasting aligned with the inactive circadian phase was associated with more favorable metabolic outcomes, highlighting circadian alignment as a key determinant of fasting-related metabolic adaptation in obesity.

## 1. Introduction

Intermittent fasting has emerged as a widely studied dietary approach with potential benefits for metabolic regulation, insulin sensitivity, and cardiometabolic health [1]. Ramadan fasting represents a unique form of intermittent fasting, characterized by daily abstinence from both food and water between dawn and sunset, with food intake restricted to the evening and pre-dawn periods. However, most human Ramadan studies are conducted under free-living conditions, in which changes in sleep patterns, dietary habits, hydration, and physical activity occur simultaneously. As a result, it remains difficult to determine whether observed metabolic effects are driven primarily by caloric intake, circadian timing of feeding, water restriction, or other lifestyle-related factors [2].

Controlled Ramadan-like fasting models in rodents provide a valuable experimental framework to isolate these variables and investigate mechanistic pathways that cannot be readily examined in humans [3,4]. Previous animal studies using Ramadan-like paradigms have reported metabolic and organ-level adaptations, supporting the utility of these models for mechanistic investigation under standardized conditions [4,5]. Nevertheless, a critical limitation of existing studies is that fasting timing and hydration status are often not experimentally disentangled. Growing evidence from time-restricted feeding studies indicates that alignment of food intake with the active circadian phase confers metabolic advantages compared with feeding during the inactive phase. In rodents, active-phase feeding has been shown to improve glucose homeostasis, insulin sensitivity, lipid metabolism, and energy expenditure, even in the absence of caloric restriction [6,7]. These effects are mediated, at least in part, by circadian regulation of peripheral metabolic pathways, including hepatic glucose metabolism, adipose tissue function, and mitochondrial energy utilization [8,9]. Importantly, most time-restricted feeding studies allow unrestricted water access, limiting their ability to model the combined food and fluid restriction characteristic of Ramadan fasting in humans.

Fasting also influences adipose tissue–derived hormones involved in appetite regulation and metabolic control. Obesity is commonly associated with hyperleptinemia, reflecting leptin resistance, and reduced adiponectin levels, while the leptin-to-adiponectin (L/A) ratio has been proposed as a sensitive marker of metabolic dysfunction and insulin resistance [10,11]. Emerging evidence indicates that adipokine secretion and sensitivity are under circadian control, regulated by both central and peripheral clocks, and are influenced by the timing of food intake relative to the active–inactive cycle [12,13]. Accordingly, fasting imposed during different circadian phases may differentially modulate leptin and adiponectin signaling, providing a mechanistic link between fasting timing and adipose-derived metabolic regulation. However, whether Ramadan-like fasting imposed during different circadian phases differentially affects adipokine profiles, and whether hydration status modifies these responses, remains unclear.

Body-weight regulation and energy balance are central to understanding fasting-induced metabolic adaptation. While fasting is generally associated with reductions in body weight and fat mass in animal models [6], accumulating evidence indicates that the timing of energy intake relative to the circadian cycle may be as important as total caloric intake. Gross energy intake (GEI) reflects total caloric consumption, whereas energy efficiency (EE) reflects how effectively consumed energy is converted into body mass. Feeding aligned with the active circadian phase enhances metabolic flexibility and energy expenditure, whereas feeding during the inactive phase favors energy storage and metabolic dysregulation [14,15,16,17]. Whether morning versus night Ramadan-like fasting differentially alters energy utilization in the context of obesity has not been systematically examined.

Electrolyte homeostasis, particularly sodium (Na^+^) and potassium (K^+^) balance, represents an additional physiological dimension of Ramadan fasting. These electrolytes play essential roles in fluid balance, neuromuscular function, and metabolic processes [15,16]. Ramadan fasting combines caloric and fluid restriction, which may influence hydration status and renal electrolyte handling. Although human studies generally report modest electrolyte changes within physiological ranges during Ramadan, most investigations focus on traditional dry fasting and do not isolate the independent contribution of water deprivation under controlled conditions [18,19,20]. Experimental comparison of dry and wet fasting models may therefore clarify whether hydration status independently modulates electrolyte balance and metabolic outcomes.

To date, no controlled experimental studies have systematically disentangled the independent effects of fasting timing and hydration status within a Ramadan-like fasting paradigm. Addressing this gap is essential for improving the mechanistic understanding of Ramadan fasting and for interpreting human observational findings.

Therefore, the present study aimed to investigate the independent and combined effects of fasting timing and hydration status on metabolic regulation in diet-induced obese rats using a controlled Ramadan-like fasting model. We compared dry and wet fasting conditions imposed during the rats’ inactive (light) versus active (dark) circadian phases. We hypothesized that fasting timing would exert a stronger influence on metabolic outcomes than hydration status. By controlling diet composition, environmental conditions, and feeding schedules, this study seeks to clarify the relative contributions of circadian phase and hydration to the metabolic effects of Ramadan-like fasting in obesity.

## 2. Materials and Methods

### 2.1. Experimental Animal

Forty-eight male Wistar Albino rats aged four weeks and weighing approximately 80 ± 10 g (range: 60–100 g) were sourced from the Experimental Surgery Animals Lab at the College of Medicine, King Saud University, Riyadh, Saudi Arabia. Animals were weight-matched prior to random allocation into experimental groups and housed in groups of eight per large cage under controlled conditions (temperature 22–24 °C and relative humidity level of 45–65%) [21].

Sample size (n = 8 rats per group) was determined based on prior rodent fasting and circadian studies demonstrating adequate power to detect medium-to-large metabolic effects [3,4,5,6,7,8,9]. Sample size estimation was informed primarily by expected differences in body weight gain and insulin sensitivity, which were defined a priori as the primary outcomes. All animals that completed the dietary induction and fasting intervention and remained in good health were included in the final analyses. No animals were excluded after randomization. Predefined exclusion criteria included signs of illness, injury, or abnormal behavior unrelated to the experimental intervention; no exclusions occurred during the study.

This study was conducted in accordance with ethical guidelines for animal research and approved by the Institutional Animal Care and Use Committee (IACUC) at King Saud University (Ref. No.: KSU-SE-24-22). All procedures prioritized animal welfare, minimized harm, and complied with local and international regulations.

### 2.2. Diets

Eight rats were fed a standard diet throughout the experiment and served as a healthy control model. Forty rats were maintained on a high-fat diet (HFD; 45.6% of total energy from fat) to induce diet-induced obesity. After 8 weeks of HFD feeding, the rats were considered obese in line with previous protocols using HFD-induced obesity in rats [19,20,22]. Both diets were obtained from Dyets Inc. (Bethlehem, PA, USA; catalog numbers 110700 for the standard diet and 104293 for the high-fat diet). The standard AIN-93G purified rodent diet provides 2.9 kcal/g, with fat accounting for about 13% of the total energy. In contrast, the modified Western-style high-fat diet provides 4.73 kcal/g, with fat accounting for approximately 45% of total energy.

### 2.3. Experimental Design

**Figure 1:**
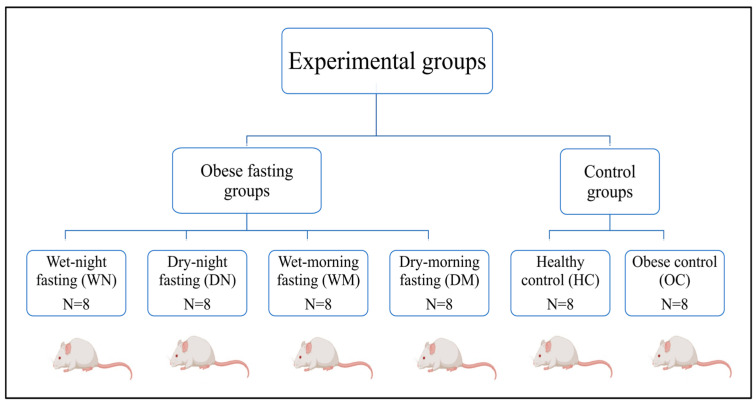
Experimental design and grouping of rats. Following obesity induction, animals were assigned to Ramadan-like fasting groups differing by fasting timing (morning, inactive phase; ~14 h fast vs. night, active phase; ~10 h fast) and hydration status (wet vs. dry), in addition to healthy and obese control groups (n = 8 per group).

The experiment was conducted over a 12-week period, consisting of 8 weeks of high-fat diet feeding to induce obesity followed by 4 weeks of the fasting intervention. Prior to the start of the experimental procedures, rats were acclimatized to the animal facility for one week with standard chow and water available ad libitum. Baseline measurements, including body weight and food and water intake, were recorded at the end of the acclimatization period. Following obesity induction, animals were randomly allocated into six experimental groups (n = 8 rats per group) using a computer-generated random number sequence. Due to the nature of the intervention, investigators were not blinded to group allocation during the experimental procedures; however, biochemical analyses were performed using standardized protocols across all groups. Animals were assigned to the following experimental groups (Figure 1):

- Group 1 (healthy control; HC): healthy rats with a 24 h ad libitum intake (standard diet + water).
- Group 2 (obese control; OC): obese rats with a 24 h ad libitum intake (HFD + water).
- Group 3 (dry morning fasting group: DM): obese rats subjected to a dry morning fasting model (no food/drinks) during light hours, followed by an ad libitum intake (HFD + water) during dark hours.
- Group 4 (wet morning fasting group WM): obese rats subjected to a wet Ramadan fasting model (no food, but free access to water) during light hours, followed by an ad libitum intake (HFD + water) during dark hours.
- Group 5 (dry night fasting group DN): obese rats subjected to a dry Ramadan fasting model (no food/drinks) during dark hours, followed by an ad libitum intake (HFD + water) during light hours.
- Group 6 (wet night fasting group WN): obese rats subjected to a wet Ramadan fasting model (no food, but free access to water) during dark hours, followed by an ad libitum intake (HFD + water) during light hours.

The Ramadan fasting model in this study refers to the application of dry fasting during the active circadian phase of rats, as described in our previous work [4]. This approach mirrors the human experience of Ramadan, where individuals fast during their biologically active daytime hours. Humans practicing Ramadan fasting consume food between dusk and dawn, following Iftar (sunset meal) and Suhoor (pre-dawn meal), which aligns eating with periods of reduced physical activity [2]. To assess the impact of this circadian alignment on metabolic outcomes, we included comparison groups that fasted during the rest (light) phase, which does not occur during human Ramadan but enables evaluation of circadian timing effects under controlled experimental conditions. Studies in both humans and animal models demonstrate that feeding during the inactive circadian phase promotes metabolic dysfunction, including impaired glucose tolerance, increased fat accumulation, and disrupted lipid metabolism [8,17,23].

Dry fasting involved the absence of both food and water, while wet fasting restricted only food, allowing water. For the night fasting groups, fasting occurred during the dark hours (rats’ active phase), while for the morning fasting groups, fasting was conducted during the light hours (rats’ inactive phase). The fasting protocol spanned 30 days, from September 1 to September 30, with lights on at 4:15 a.m. and lights off at 6:12 p.m. Although fasting duration differed between groups (approximately 14 h for morning fasting and 10 h for night fasting), this design was intentional to preserve the ecological validity of Ramadan-like fasting. Nevertheless, this unequal duration represents a limitation of the study, as the effects of fasting timing cannot be fully disentangled from fasting duration within this experimental framework. Recent research comparing different time-restricted feeding windows suggests that timing may be more important than duration for metabolic outcomes [6,9,24].

During the fasting period (weeks 9–12), food and water intake were measured by providing precise amounts of food and water, then calculating the leftovers after each fasting interval.

### 2.4. Body Weight and Food Intake

BW was recorded weekly using an EK4150 digital scale (Etekcity, Anaheim, CA, USA). Weekly weight gain was calculated by subtracting each week’s mean BW from the following week’s value. Food intake was measured weekly on a calibrated precision scale (accuracy: 0.01 mg) by subtracting leftover diet from the initial amount provided. To estimate gross energy intake (GEI), we multiplied total weekly food intake per cage by the diet’s energy density (4.8 kcal/g), then normalized these values per rat and converted them to kcal/rat/day. Energy efficiency (EE) was expressed as the ratio of weekly weight gain (g) to weekly GEI (kcal), indicating how many g were gained per kcal consumed. We also calculated cumulative GEI and EE over the entire 4-week fasting period to assess overall metabolic adaptation [22].

Food and water intake were recorded at the cage level; therefore, the cage was considered the experimental unit for intake-related variables. As a result, GEI and EE were analyzed descriptively only, without inferential statistical testing. In contrast, body weight and biochemical outcomes were analyzed at the individual rat level.

### 2.5. Urine Collection

Urine samples were collected during the final week of the intervention using individual metabolic cages over a 24 h period. Collected urine was centrifuged to remove debris and stored at −80 °C until subsequent biochemical analysis.

### 2.6. Animal Anesthesia and Blood Sampling

All animals underwent overnight fasting prior to anesthesia, which was administered intraperitoneally using ketamine (90 mg/kg) and xylazine (10 mg/kg) as described by Mete et al. [25]. All terminal blood samples were collected during the light phase to minimize circadian-related variability. Approximately 1 mL of blood was drawn via cardiac puncture into plain collection tubes. These tubes were then centrifuged at 3000 rpm for 10 min at room temperature to separate the serum. The serum was carefully transferred to fresh tubes and stored at −80 °C for further biochemical analyses. Following blood collection, euthanasia was performed using cervical dislocation.

### 2.7. Biochemical Analysis

All ELISA assays were validated for precision and accuracy according to manufacturer specifications. Fasting blood glucose was measured in the morning using a rat-appropriate handheld glucometer (AlphaTRAK 3, Zoetis Inc., Parsippany, NJ, USA) via tail-vein sampling after an overnight fast. Serum glucose and insulin concentrations were quantified using rat-specific ELISA kits (Glucose: Cat. MBS7233226; Insulin: Cat. MBS8801371; MyBioSource, San Diego, CA, USA). The glucose ELISA demonstrated intra- and inter-assay coefficients of variation (CVs) <10% and <12%, respectively, with a validated analytical sensitivity of 0.1 mmol/L and spike-recovery values of 94–103%. The insulin assay showed a detection range of 15.63–1000 pg/mL and a sensitivity of 4.93 pg/mL, with intra- and inter-assay CVs <10% and <12%, respectively. All glucose and insulin values fell within the validated detection limits; samples above the upper quantification limit (ULOQ) were appropriately diluted and re-assayed. The homeostasis model assessment of insulin resistance (HOMA-IR) was calculated as fasting insulin (ng/mL) × fasting glucose (mmol/L)/22.5.

Lipid profiles, including total cholesterol (TC), triglycerides (TG), high-density lipoprotein (HDL), and low-density lipoprotein (LDL), were assessed using ELISA kits (MyBioSource; Cat. MBS8807820; Cat. MBS8807309; Cat. MBS266554; Cat. MBS2508037; respectively). The TC assay demonstrated a sensitivity of 2.63 μg/mL and a detection range of 7.82–500 μg/mL, while the TG assay showed a sensitivity of 1.6 μg/mL and a detection range of 15.63–1000 μg/mL. HDL ELISA had a detection range of 200–3.12 ng/mL with a minimum detectable concentration of 0.6 ng/mL; intra- and inter-assay CVs were ≤8% and ≤12%, respectively. LDL ELISA demonstrated a sensitivity of 0.1 μg/mL and a detection range of 0.16–10 μg/mL, with repeatability (CV) < 10%. All lipid measurements fell within validated analytical ranges, and samples exceeding the ULOQ were diluted and re-assayed.

Serum leptin and adiponectin were quantified using ELISA kits (MyBioSource; Cat. MBS2884341 and Cat. MBS703720, respectively). The Leptin assay demonstrated a detection range of 0.156–10 ng/mL with a sensitivity < 0.056 ng/mL and intra- and inter-assay CVs of ≤3.5% and ≤7.2%, respectively. Adiponectin ELISA had a detection range of 0.156–10 ng/mL, a minimum detectable level of < 0.039 ng/mL, and intra- and inter-assay CVs of <8% and <10%, respectively. All values were within validated assay limits; samples above the ULOQ were diluted and repeated. The Leptin-to-adiponectin (L/A) ratio was calculated by dividing serum leptin (ng/mL) by adiponectin (ng/mL) as an index of adipokine balance.

Serum and urine Na^+^ and K^+^ concentrations were measured using rat-specific ELISA kits (MyBioSource; Cat. MBS3808460 and Cat. MBS3808979, respectively). The Na^+^ assay demonstrated a sensitivity of 1 mmol/L, an assay range of 7.5–240 mmol/L, and intra- and inter-assay CVs < 15%. The K^+^ assay showed a sensitivity of 0.1 mmol/L, an assay range of 0.5–16 mmol/L, and intra- and inter-assay CVs < 15%. All electrolyte values fell within validated detection ranges, and samples above the ULOQ were diluted and re-assayed.

Serum insulin, leptin, and adiponectin concentrations were measured in n = 6 rats per group due to limited sample volume. This reduced sample size was predefined prior to statistical analysis. Urinary electrolyte measurements were performed in a subset of animals (n = 3 rats per group) as an exploratory pilot analysis due to limited urine availability, and this reduced sample size was also predefined prior to statistical analysis.

Outcome measures were predefined to guide interpretation in the context of multiple testing. Primary outcomes included body-weight gain, fasting insulin, and HOMA-IR, as these directly reflect metabolic adaptation to fasting. Secondary or exploratory outcomes included adipokines (leptin, adiponectin, and the leptin/adiponectin ratio), serum electrolytes, and urinary electrolytes. Exploratory outcomes were analyzed to generate mechanistic insights and should be interpreted cautiously.

All biochemical measurements were performed in duplicate, and mean values were used for statistical analysis to enhance accuracy and reproducibility. Procedures and measurements were performed using the same protocols across groups. Measurements were conducted at consistent time points to reduce variability.

### 2.8. Statistical Analysis

Primary outcomes were defined a priori as body-weight gain, fasting insulin, and HOMA-IR. Secondary outcomes included fasting glucose (measured by glucometer and serum assay), lipid profile parameters, and serum electrolyte concentrations (Na^+^ and K^+^). Exploratory outcomes included adipokines (leptin, adiponectin, and L/A ratio) and urinary electrolyte measurements; these were analyzed to generate mechanistic insight, were based on reduced sample sizes, and were interpreted cautiously in the context of multiple testing.

Statistical analyses were conducted using GraphPad Prism version 7.1 (GraphPad Software Inc., San Diego, CA, USA). Data are presented as mean ± standard deviation (SD). For primary outcomes, 95% confidence intervals for group means were calculated using the t-distribution based on the corresponding degrees of freedom. Normality was assessed using the Shapiro–Wilk test. Most outcomes were obtained from eight rats per group; however, adipokines (leptin and adiponectin) were measured in six rats per group due to limited serum volume, and urinary electrolytes (Na^+^, K^+^) were evaluated in three rats per group as a pilot assessment. Analyses were performed using complete-case data with no imputation for missing values.

Group differences were examined using one-way ANOVA followed by Tukey’s post hoc test for multiple comparisons. For outcomes influenced by BW (lipid profile parameters), an analysis of covariance (ANCOVA) was applied with BW included as a covariate to obtain adjusted group means and corrected post hoc comparisons. Multiple metabolic endpoints were examined without formal adjustment for multiplicity; therefore, secondary findings should be interpreted with caution. All statistical tests were two-sided. Statistical significance was defined as *p* < 0.05, and exact *p*-values are reported in the text and figures where applicable.

Food and water intake were recorded descriptively at the cage level rather than the individual rat level. Consequently, GEI and EE were summarized descriptively without inferential statistical testing, and differences among groups are described as observed trends only.

## 3. Results

### 3.1. Body Weight

**Figure 2:**
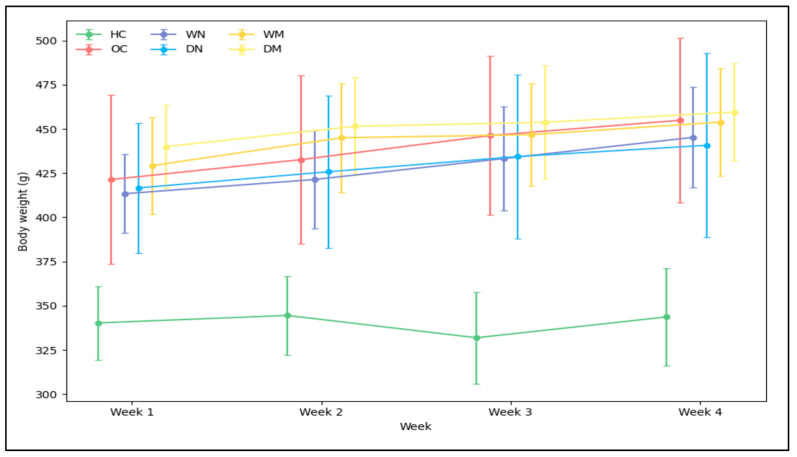
Weekly body weight changes in healthy control (HC), obese control (OC), wet-night fasting (WN), dry-night fasting (DN), wet-morning fasting (WM), and dry-morning fasting (DM) groups over the 4-week intervention period. Data are presented as mean ± SD (n = 8 rats per group). Slight horizontal offsets were applied to improve the visualization of group variability at each time point. For the total 4-week body-weight gain, the corresponding 95% confidence intervals were: OC (27.5–39.5 g), WN (24.1–39.7 g), DN (5.4–42.9 g), WM (13.6–35.6 g), and DM (13.4–25.9 g).

Analysis of individual BW data revealed a significant group effect on weight gain over the 4-week intervention period (one-way ANOVA, F (5,42) = 10.72, *p* < 0.0001). The group effect was large in magnitude (partial η^2^ = 0.56), indicating that the fasting condition explained a substantial proportion of the variance in body-weight gain. Both the obese control (OC) and wet night fasting (WN) groups exhibited the greatest weight gain and were significantly higher than the healthy control (HC) group (*p* < 0.001). Intermediate increases were observed in the dry night (DN) and wet morning (WM) groups. Notably, the DN group exhibited greater inter-individual variability in body-weight gain compared with the other fasting groups, indicating heterogeneous responses to night fasting. Among the obese fasting groups, the dry morning (DM) group showed the smallest weight gain, which was significantly lower than OC and WN (*p* < 0.05). All obese groups gained significantly more BW than HC (Figure 2).

### 3.2. Food and Water Intake

Food and water intake varied across experimental groups (Table 1). The WM group exhibited the highest average food intake, whereas the WN group consistently consumed the least. Water intake was higher in wet fasting groups compared with dry fasting groups, as expected based on hydration availability, with the highest intake observed in the WN group and the lowest in the DM group.

Across the intervention period, gross energy intake (GEI) was comparable among obese groups, ranging from 1828.7 kcal (WN) to 2181.7 kcal (WM) per rat. Despite similar GEI, weight gain differed markedly between groups, suggesting differences in energy utilization rather than caloric intake per se, with WN and OC showing the greatest increases and DM the lowest. Energy efficiency (EE) followed a similar pattern, with the highest values observed in WN and the lowest in DM (Table 2). As food and water intake were measured at the cage level, GEI and EE are presented descriptively without inferential statistical testing.

### 3.3. Blood Analysis

#### 3.3.1. Glucose

Serum glucose concentrations differed significantly among groups (one-way ANOVA, F(5,42) = 5.82, *p* = 0.00036), with a large effect size (partial η^2^ = 0.41). Post hoc analysis showed that the OC group and the night fasting groups (WN and DN) had significantly higher serum glucose levels compared with the HC group (*p* < 0.05 for all). In contrast, serum glucose levels in both morning fasting groups (WM and DM) did not differ significantly from HC (*p* > 0.05).

**Figure 3:**
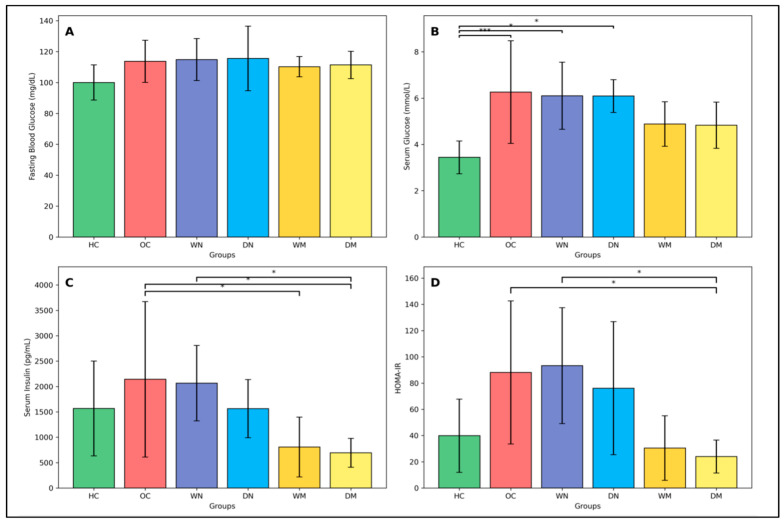
Fasting blood glucose, serum glucose, serum insulin, and insulin resistance across experimental groups. (**A**) Fasting blood glucose measured using a tail-vein handheld glucometer (mg/dL). (**B**) Serum glucose measured after cardiac puncture (mmol/L). (**C**) Serum insulin concentrations (pg/mL). (**D**) Insulin resistance assessed by the homeostatic model assessment (HOMA-IR). Data are presented as mean ± SD. Fasting blood glucose and serum glucose were analyzed using n = 8 rats per group, whereas serum insulin and HOMA-IR were analyzed using n = 6 rats per group. Horizontal black brackets indicate significant pairwise comparisons (* *p* < 0.05; *** *p* < 0.001). For primary metabolic outcomes, corresponding 95% confidence intervals were as follows: serum insulin—HC (586–2546), OC (535–3749), WN (1287–2845), DN (964–2164), WM (188–1426), DM (394–991); HOMA-IR—HC (10.6–69.1), OC (30.8–145.4), WN (47.0–139.6), DN (22.8–129.4), WM (4.6–56.2), DM (10.8–37.1).

Fasting blood glucose measured by tail-vein glucometer did not differ significantly among groups (one-way ANOVA, F(5,42) = 1.50, *p* = 0.21) and showed only a small-to-moderate effect size (partial η^2^ = 0.15), despite slightly higher mean values in OC and fasting groups compared with HC. (Figure 3).

#### 3.3.2. Insulin and Insulin Resistance (HOMA-IR)

Serum insulin levels differed significantly among groups (one-way ANOVA, F(5,30) = 2.98, *p* = 0.027), with a moderate-to-large effect size (partial η^2^ = 0.33). OC and WN groups exhibited the highest insulin levels, whereas both morning fasting groups (WM and DM) showed markedly lower concentrations. Post hoc analysis confirmed significantly lower insulin levels in WM and DM compared with OC, and in DM compared with WN (*p* < 0.05).

HOMA-IR values also differed significantly across groups (one-way ANOVA, F(5,30) = 3.76, *p* = 0.0092), with a large effect size (partial η^2^ = 0.39). OC, WN, and DN exhibited higher insulin resistance indices, whereas both morning fasting groups showed the lowest values. Post hoc analysis confirmed significantly lower HOMA-IR in DM compared with OC and WN (*p* < 0.05), while no other pairwise comparisons reached statistical significance (Figure 3).

#### 3.3.3. Lipid Profile

After adjustment for body weight using ANCOVA, significant group effects were observed for total cholesterol (TC; *p* = 0.0011), triglycerides (TG; *p* = 0.0001), LDL (*p* = 0.0479), and HDL (*p* = 0.034). OC exhibited the highest TC and TG levels, whereas both morning fasting groups (WM and DM) showed significantly lower TC compared with OC. TG levels were significantly reduced in all fasting groups relative to OC.

**Figure 4:**
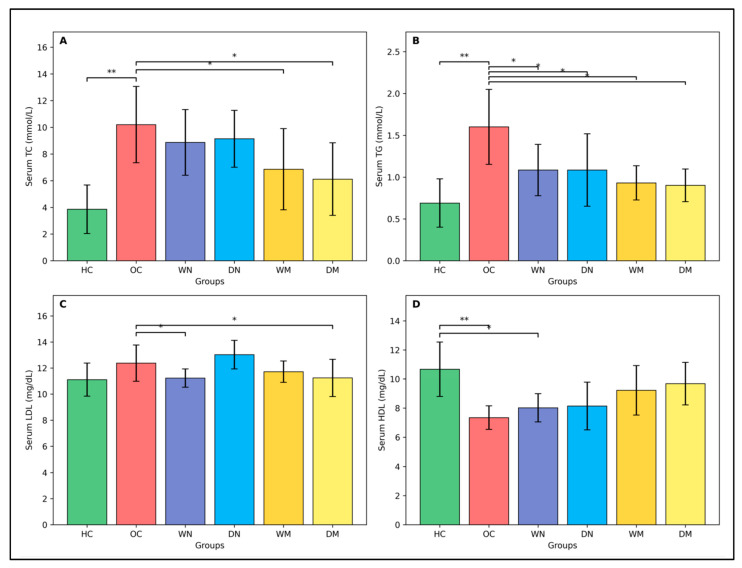
Serum lipid profile across experimental groups. (**A**) Total cholesterol (TC, mmol/L), (**B**) triglycerides (TG, mmol/L), (**C**) low-density lipoprotein cholesterol (LDL, mg/dL), and (**D**) high-density lipoprotein cholesterol (HDL, mg/dL). Data are presented as mean ± SD (n = 8 rats per group). Group differences were analyzed using analysis of covariance (ANCOVA) with body weight included as a covariate, followed by Tukey’s post hoc test for multiple comparisons. Horizontal brackets indicate statistically significant pairwise differences (* *p* < 0.05, ** *p* < 0.01). Abbreviations: HC, healthy control; OC, obese control; WN, wet-night fasting; DN, dry-night fasting; WM, wet-morning fasting; DM, dry-morning fasting.

LDL levels were highest in OC and significantly greater than WN and DM, whereas HDL levels were highest in HC and lowest in OC. No significant differences in HDL were detected between the OC and fasting groups. Overall, after adjustment for body weight, TC and TG levels were lower in the morning fasting groups compared with the OC group, whereas HDL levels showed more limited between-group differences (Figure 4).

#### 3.3.4. Adipokines

Adipokine analyses were exploratory and conducted in a reduced sample size (n = 6 per group); therefore, results should be interpreted cautiously in the context of interindividual variability. Serum leptin levels varied significantly among groups (*p* < 0.05, one-way ANOVA). Tukey’s HSD post hoc analysis showed that the OC group had significantly higher leptin levels than both morning fasting groups (WM and DM) (*p* = 0.042 and *p* = 0.049, respectively). In addition, the WN group exhibited significantly higher leptin concentrations compared with the HC group (*p* = 0.021). No other pairwise comparisons reached statistical significance (*p* > 0.05) (Table 3).

Serum adiponectin levels differed significantly among groups (one-way ANOVA, *p* < 0.01). Post hoc analysis revealed that adiponectin levels were significantly higher in the HC group compared with the OC and DM groups (*p* = 0.003 and *p* = 0.002, respectively). The WM group also exhibited significantly higher adiponectin concentrations than OC and DM (*p* = 0.024 and *p* = 0.018, respectively). No other pairwise differences were statistically significant (*p* > 0.05). Notably, among fasting groups, only the WM group consistently exhibited leptin, adiponectin, and L/A ratio values approaching those of the healthy control group, whereas greater variability limited confident interpretation of results in other fasting conditions (Table 3).

**Figure 5:**
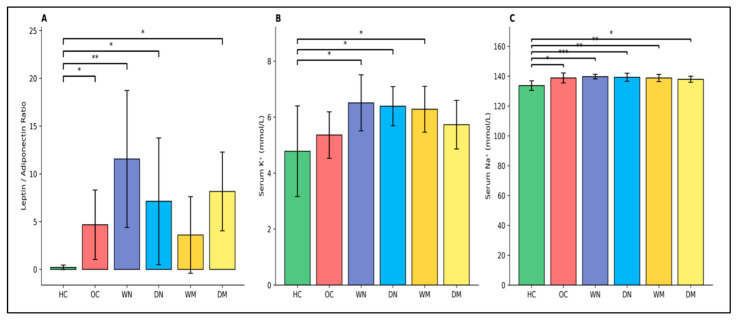
Leptin/adiponectin ratio and serum electrolyte levels across experimental groups. (**A**) Leptin/adiponectin (L/A) ratio (n = 6 rats per group), (**B**) serum potassium (K^+^, mmol/L; n = 8 rats per group), and (**C**) serum sodium (Na^+^, mmol/L; n = 8 rats per group). Data are presented as mean ± SD. Group differences were analyzed using one-way ANOVA followed by Tukey’s post hoc test. Horizontal brackets indicate statistically significant pairwise comparisons (* *p* < 0.05, ** *p* < 0.01, *** *p* < 0.001). Abbreviations: HC, healthy control; OC, obese control; WN, wet-night fasting; DN, dry-night fasting; WM, wet-morning fasting; DM, dry-morning fasting.

The leptin/adiponectin (L/A) ratio differed significantly across groups (one-way ANOVA, *p* < 0.05). Tukey’s HSD post hoc test demonstrated that the L/A ratio was significantly higher in the OC, WN, DN, and DM groups compared with the HC group (*p* = 0.030, *p* = 0.006, *p* = 0.028, and *p* = 0.014, respectively). No significant difference in the L/A ratio was observed between the HC and WM groups, and no other pairwise comparisons reached statistical significance (*p* > 0.05) (Figure 5A).

#### 3.3.5. Electrolytes

##### Serum Potassium and Sodium

Serum potassium (K^+^) and sodium (Na^+^) levels differed significantly among groups (*p* = 0.003 and *p* = 0.00043, respectively). HC exhibited the lowest K^+^ and Na^+^ concentrations, whereas all obese groups showed modestly higher values within physiological ranges. No significant differences were detected among obese fasting groups, indicating preserved electrolyte homeostasis despite differences in fasting timing and hydration (Figure 5B,C).

##### Potassium and Sodium in Urine (Exploratory Pilot Analysis)

Urinary Na^+^ and K^+^ excretion showed considerable variability among groups (n = 3 per group). Due to the small sample size and exploratory nature of this analysis, no statistical testing was performed, and no firm conclusions can be drawn regarding fasting-related differences in urinary electrolyte handling (Table 3).

## 4. Discussion

This study investigated the relative contributions of fasting timing (morning versus night) and hydration status (wet versus dry) to metabolic regulation in diet-induced obese rats using a controlled Ramadan-like fasting model. Across outcomes, a consistent pattern emerged: fasting aligned with the inactive circadian phase (morning fasting) was associated with more favorable metabolic profiles than fasting during the active phase, whereas hydration status exerted more modest effects. These findings reinforce the concept that circadian alignment of feeding and fasting exerts a dominant influence on metabolic regulation, potentially outweighing hydration status in shaping cardiometabolic outcomes [26,27]. Conceptually, these results support a hierarchical model in which circadian timing of fasting acts as the primary driver of metabolic adaptation in obesity, while hydration status functions as a secondary physiological modulator. Within this framework, alignment of fasting with the inactive circadian phase improves glucose–insulin regulation, lipid handling, and adipose signaling, whereas hydration status mainly influences fluid and electrolyte balance without fundamentally altering the core metabolic trajectory.

Body weight measurements supported this interpretation. Obese control (OC) and night-fasting groups exhibited the greatest weight gain, whereas morning-fasting groups—particularly the dry-morning (DM) group—showed attenuated weight gain despite comparable or, in some cases, lower estimated energy intake. Experimental and mechanistic studies indicate that feeding during an inappropriate circadian phase suppresses thermogenesis, alters substrate utilization, and promotes lipid storage, thereby reducing metabolic flexibility [26,27,28]. In addition, Ramadan fasting has been shown to remodel the gut microbiome in humans, potentially influencing energy harvest and metabolic signaling; however, microbiota composition was not assessed in the present study [29].

The fasting durations differed between morning (14 h) and night (10 h) groups, reflecting the natural photoperiod in Riyadh during the study period and prioritizing ecological validity. Ramadan fasting duration varies widely by season and latitude in humans, ranging from approximately 11 to over 18 h [26]. Recent evidence suggests that meal timing relative to circadian phase may exert greater metabolic influence than fasting duration alone [6,9,24]. The present findings are consistent with this framework, as morning-fasting groups demonstrated more favorable glucose–insulin and lipid profiles despite longer fasting intervals, suggesting that circadian alignment outweighed fasting duration in determining metabolic outcomes.

Alterations in glucose regulation observed in this study are consistent with the growing body of evidence highlighting the importance of circadian alignment in metabolic control. Elevated serum glucose in obese and night-fasting conditions, contrasted with values closer to the healthy range under morning fasting, supports the concept that the timing of fasting relative to the circadian cycle modulates glycemic homeostasis in obesity. Experimental and clinical studies indicate that feeding–fasting schedules aligned with the circadian phase influence hepatic glucose output and peripheral glucose utilization through clock-regulated metabolic pathways [27,28]. The absence of significant differences in fasting blood glucose measured by a point-of-care glucometer, despite clear group differences in serum glucose, likely reflects methodological differences related to sampling site, analytical approach, and timing of measurement relative to terminal blood collection. [26]. Together, these observations underscore the importance of circadian timing in shaping glucose responses to fasting while highlighting the need for cautious interpretation of method-dependent glucose readouts.

Alterations in insulin dynamics provided the clearest indication. Patterns observed across insulin and insulin-resistance indices are consistent with enhanced glucose–insulin homeostasis when fasting is aligned with the inactive circadian phase, whereas fasting during the active phase appears less effective in mitigating obesity-associated insulin resistance. This interpretation aligns with the ‘metabolic switch’ framework, in which fasting-induced improvements in insulin sensitivity and metabolic flexibility are modulated by circadian regulation of peripheral clocks and nutrient-sensing pathways [26,28,30].

Among lipid fractions, LDL emerged as the most selectively responsive parameter to fasting timing in this model, accompanied by reductions in total cholesterol and triglycerides, while HDL showed minimal between-group variation. Lipid-related outcomes further support the central role of fasting timing in shaping metabolic adaptation. Rather than uniformly affecting all lipid fractions, fasting appeared to exert selective effects on cholesterol handling, with a more pronounced influence on atherogenic lipids than on HDL. This pattern is consistent with circadian regulation of lipid metabolism, whereby clock-controlled enzymes govern cholesterol transport, fatty acid oxidation, and lipoprotein turnover in a phase-dependent manner [27,28,31,32,33]. Circadian disruption has been shown to preferentially impair LDL handling and lipid rhythmicity, rather than globally improving lipid profiles. In the context of Ramadan fasting, previous work suggests that meal timing and structure within the feeding window—particularly avoidance of late, large meals—may be critical for preserving lipid metabolic alignment [26,34].

Given the reduced sample size and inter-individual variability, adipokine findings should be interpreted cautiously. Within this context, analyses revealed group differences in leptin, adiponectin, and the leptin/adiponectin ratio, reflecting variations in adipose tissue signaling across fasting conditions. Elevated L/A ratios in OC and WN groups are consistent with impaired adipose–metabolic signaling. Importantly, lower leptin levels in morning fasting groups may reflect improved leptin sensitivity rather than reduced adiposity alone, as fasting and circadian alignment can modulate hypothalamic signaling and inflammatory tone [10,11,30,35]. Adiponectin’s insulin-sensitizing effects may also depend on circadian-regulated receptor expression and downstream pathways, providing a mechanistic framework for the observed adipokine patterns [26,28,36].

Hydration effects were most evident in expected differences in water intake and in electrolyte handling. Serum Na^+^ differed statistically between healthy and obese conditions but remained within a narrow physiological range across obese conditions, reflecting preserved renal and hormonal homeostatic regulation. Similar preservation of serum and urinary osmolality during daytime dry fasting has been reported in controlled human studies, suggesting activation of adaptive renal mechanisms that maintain electrolyte balance despite fluid restriction [37]. K^+^ levels showed modest elevations in OC and fasting groups, consistent with shifts in renal handling and intracellular–extracellular distribution rather than overt electrolyte dysregulation. These findings support the view that hydration status acts as a secondary but physiologically relevant modulator, influencing electrolyte balance and potentially renal and hormonal responses during fasting [15,16,26]. Urinary electrolyte observations should be interpreted cautiously, as they were derived from an exploratory pilot analysis with a limited sample size and are intended to complement, rather than extend, the serum electrolyte findings. Overall, these findings indicate that hydration status contributes to physiological adaptation during fasting, particularly through fluid and electrolyte regulation, but does not supersede circadian timing as the primary determinant of metabolic outcomes in this model.

Translating these findings to human Ramadan fasting requires acknowledging species differences in circadian biology. Rats are nocturnal, whereas humans are diurnal; therefore, morning fasting in this model corresponds to fasting during the inactive (light) phase in rats, in contrast to human Ramadan fasting, which occurs during the active daytime period. However, the principle of circadian alignment remains relevant. Human intervention studies demonstrate that aligning food intake with the active circadian phase (early time-restricted feeding) improves insulin sensitivity, glucose tolerance, and cardiometabolic markers, even in the absence of weight loss [24,38]. Despite the inversion of light–dark activity cycles between nocturnal rodents and diurnal humans, the underlying metabolic principle remains consistent: fasting during the inactive/rest phase and feeding during the active phase appear to confer the greatest metabolic benefit. An additional consideration is that rats in this study had ad libitum access to food during feeding windows, whereas humans during Ramadan typically consume structured meals. Meal frequency and eating patterns may independently influence metabolic outcomes, as continuous grazing can affect insulin secretion patterns and metabolic efficiency differently than discrete meals [39,40]. During Ramadan, Muslims eat between dusk and dawn, with two main meals: Iftar (the sunset meal) and Suhoor (the pre-dawn meal). This biphasic eating pattern generates two cortisol peaks that help synchronize both central and peripheral circadian clocks [26,41]. When these meals occur at their traditional times, Iftar shortly after sunset and Suhoor close to dawn, the feeding window aligns with reduced physical activity and supports metabolic regulation [39]. However, modern Ramadan practices often involve staying awake late after Iftar, consuming multiple meals and snacks throughout the night, and delaying sleep, which may disrupt circadian alignment and diminish metabolic benefits [26,34]. Applied to human Ramadan fasting, this supports recommendations to consume the two main meals near the beginning (dusk) and end (dawn) of the feeding window, avoid nocturnal grazing, and prioritize adequate nighttime sleep to maintain clock synchronization [40,42]. Transforming the nighttime feeding window into prolonged or continuous eating periods may counteract the metabolic benefits associated with structured Ramadan fasting, as frequent nocturnal food intake can disrupt peripheral clock gene expression, impair glucose tolerance, and promote lipid accumulation independent of total caloric intake [26,39]. Whether water availability during fasting hours influences human metabolic adaptation remains unknown, though electrolyte homeostasis appears well-preserved under controlled dry fasting conditions. Thus, hydration status appears to modulate physiological adaptation to fasting—particularly fluid and electrolyte handling—without fundamentally altering the core metabolic trajectory driven by circadian timing.

Finally, the absence of direct mechanistic measurements highlights key directions for future research. Restricted feeding is known to modulate peripheral clock gene expression and nutrient-sensing pathways in rats, and Ramadan literature emphasizes roles for autophagy, gene regulation, and gut microbiota rhythmicity in fasting-related metabolic adaptation [28,29,35]. Incorporating these endpoints in future studies would strengthen the mechanistic understanding of how fasting timing shapes metabolic health. Future studies should also examine sex-specific responses to fasting timing, given known differences in circadian regulation and metabolic adaptation between males and females. Longer-term interventions are needed to determine whether the metabolic benefits associated with circadian-aligned fasting are sustained over time.

Several limitations of the current study should be acknowledged. Food and water intake were measured at the cage level rather than individually; therefore, the cage was considered the experimental unit for intake-related variables, and GEI and EE were analyzed descriptively without inferential statistics. This approach reduces precision at the individual level and limits causal interpretation of energy balance outcomes, which should be interpreted cautiously. Sample sizes were limited for some outcomes, particularly adipokines (n = 6 per group) and urinary electrolytes (n = 3 per group), restricting statistical power and rendering these findings exploratory. The intervention duration was limited to four weeks, which may not capture long-term metabolic adaptations. In addition, fasting duration differed between morning and night fasting groups as a result of the circadian light–dark cycle, reflecting real-world Ramadan conditions but limiting the ability to fully disentangle the independent effects of fasting timing from fasting duration. Mechanistic pathways were not directly assessed; clock gene expression, brown adipose tissue activity, gut microbiota composition, and autophagy markers were not measured. Only male rats were studied, precluding evaluation of sex-specific circadian responses. Locomotor activity was not directly measured in this study; therefore, potential contributions of physical activity to the observed metabolic changes cannot be excluded. Future studies incorporating activity monitoring would help clarify this relationship. Finally, translational interpretation requires caution because rats are nocturnal, and fasting timing in this model does not directly correspond to human Ramadan fasting physiology, despite shared circadian principles.

Despite these limitations, this study provides the first controlled experimental evidence that circadian timing of fasting is a dominant determinant of metabolic adaptation in obesity, exerting stronger effects than hydration status across key metabolic outcomes.

## 5. Conclusions

This study demonstrates that fasting timing exerts a stronger influence on metabolic regulation in obese rats than hydration status. Fasting aligned with the inactive circadian phase was associated with reduced body-weight gain, lower insulin levels, improved insulin sensitivity, and a more favorable lipid profile compared with fasting during the active phase, despite comparable energy intake. In contrast, hydration status primarily affected water intake and electrolyte handling and did not substantially modify core metabolic outcomes. Importantly, in this study, “morning fasting” corresponds to fasting during the inactive (light) circadian phase in rats, whereas “night fasting” corresponds to fasting during the active (dark) phase.

Unlike previous Ramadan and intermittent fasting studies, this work directly isolates the independent effects of fasting timing and hydration status within a controlled experimental model. By experimentally disentangling fasting timing from hydration under controlled Ramadan-like conditions, these findings highlight circadian alignment of the feeding–fasting cycle as a key determinant of metabolic adaptation to fasting, with hydration acting as a secondary physiological modulator. Translationally, these results support the importance of structured meal timing during Ramadan, with food intake concentrated near sunset and pre-dawn and avoidance of prolonged nocturnal eating, to preserve circadian metabolic alignment.

## Figures and Tables

**Table 1:** Food and water intake across experimental groups (descriptive analysis).

Variable	OC	WN	DN	WM	DM
Food intake (g/week/group)	919.75 ± 34.9	773.25 ± 82.8	786.25 ± 92.5	922.50 ± 206.6	873.25 ± 88.4
Water intake (mL/week/group)	1601.00 ± 148.6	1762.25 ± 135.4	1203.75 ± 323.8	1585.75 ± 337.2	1350.50 ± 369.7

Note: Values are expressed as mean ± SD (n = 8 rats per group). Food and water intake were recorded at the cage level, and therefore the cage was considered the experimental unit. No inferential statistical analyses were performed for intake variables; values are presented for descriptive purposes only (see Methods, Section 2.7).

**Table 2:** Gross energy intake, body-weight change, and energy efficiency across experimental groups (descriptive analysis).

Group	Total GEI (kcal/Rat)	BW Change (g)	Energy Efficiency (g/kcal)
OC	2175.2	33.50 ± 7.21	0.0123
WN	1828.7	31.88 ± 9.30	0.0174
DN	1859.5	24.12 ± 22.39	0.0130
WM	2181.7	24.63 ± 13.16	0.0113
DM	2065.2	19.63 ± 7.46	0.0094

Note: Values represent per-rat estimates derived from cage-level food intake (total weekly food intake × 4.8 kcal/g, divided by the number of rats per cage). Gross energy intake (GEI) and energy efficiency (EE) were analyzed descriptively only, and no inferential statistical testing was performed because the cage was the experimental unit (see Methods, Section 2.7).

**Table 3:** Serum adipokines and urinary electrolyte excretion across experimental groups.

Variable	HC	OC	WN	DN	WM	DM	*p*-Value
Serum leptin (ng/mL)	0.09 ± 0.09	0.66 ± 0.35 ^ab^	1.78 ± 1.14	1.51 ± 0.85	1.02 ± 0.96 ^a^	1.07 ± 0.48 ^b^	0.007
Serum adiponectin (ng/mL)	0.57 ± 0.18 ^a^	0.16 ± 0.09 ^b^	0.18 ± 0.11	0.31 ± 0.16	0.44 ± 0.28 ^ab^	0.14 ± 0.04 ^b^	0.0002
Urinary K^+^ (mmol/kg/day)	4.85 ± 0.40	9.12 ± 0.99	7.29 ± 2.46	10.33 ± 1.59	11.31 ± 1.12	5.35 ± 2.38	Exploratory
Urinary Na^+^ (mmol/kg/day)	18.01 ± 6.10	16.52 ± 2.00	16.40 ± 1.54	13.65 ± 1.90	11.99 ± 2.58	15.65 ± 3.14	Exploratory

Note: Values are expressed as mean ± SD. Serum leptin and adiponectin were measured in n = 6 rats per group. Urinary electrolyte excretion was assessed in a subset of animals (n = 3 rats per group) and is presented for exploratory purposes only; no inferential statistical testing was performed for urinary electrolytes. For serum adipokines, different superscript letters within the same row indicate statistically significant differences between groups (one-way ANOVA followed by Tukey’s post hoc test, *p* < 0.05).

## Data Availability

The data presented in this study are available from the corresponding author upon reasonable request due to ethical and institutional restrictions related to animal experimentation.

## Abbreviations

The following abbreviations are used in this manuscript: HFD, high-fat diet; BW, body weight; HC, healthy control; OC, obese control; DM, dry morning fasting group; WM, wet morning fasting group; DN, dry night fasting group; WN, wet night fasting group; TC, total cholesterol; TG, triglycerides; HDL, high-density lipoprotein; LDL, low-density lipoprotein; SIRT1, sirtuin 1; SIRT3, sirtuin 3; Na^+^, sodium; K^+^, potassium; EE, energy efficiency; GEI, gross energy intake; L/A ratio, leptin/adiponectin ratio.
